# Changes in Influenza and Other Respiratory Virus Activity During the COVID-19 Pandemic — United States, 2020–2021

**DOI:** 10.15585/mmwr.mm7029a1

**Published:** 2021-07-23

**Authors:** Sonja J. Olsen, Amber K. Winn, Alicia P. Budd, Mila M. Prill, John Steel, Claire M. Midgley, Krista Kniss, Erin Burns, Thomas Rowe, Angela Foust, Gabriela Jasso, Angiezel Merced-Morales, C. Todd Davis, Yunho Jang, Joyce Jones, Peter Daly, Larisa Gubareva, John Barnes, Rebecca Kondor, Wendy Sessions, Catherine Smith, David E. Wentworth, Shikha Garg, Fiona P. Havers, Alicia M. Fry, Aron J. Hall, Lynnette Brammer, Benjamin J. Silk

**Affiliations:** ^1^Influenza Division, National Center for Immunization and Respiratory Diseases, CDC; ^2^Division of Viral Diseases, National Center for Immunization and Respiratory Diseases, CDC.

The COVID-19 pandemic and subsequent implementation of nonpharmaceutical interventions (e.g., cessation of global travel, mask use, physical distancing, and staying home) reduced transmission of some viral respiratory pathogens (*1*). In the United States, influenza activity decreased in March 2020, was historically low through the summer of 2020 (*2*), and remained low during October 2020–May 2021 (<0.4% of respiratory specimens with positive test results for each week of the season). Circulation of other respiratory pathogens, including respiratory syncytial virus (RSV), common human coronaviruses (HCoVs) types OC43, NL63, 229E, and HKU1, and parainfluenza viruses (PIVs) types 1–4 also decreased in early 2020 and did not increase until spring 2021. Human metapneumovirus (HMPV) circulation decreased in March 2020 and remained low through May 2021. Respiratory adenovirus (RAdV) circulated at lower levels throughout 2020 and as of early May 2021. Rhinovirus and enterovirus (RV/EV) circulation decreased in March 2020, remained low until May 2020, and then increased to near prepandemic seasonal levels. Circulation of respiratory viruses could resume at prepandemic levels after COVID-19 mitigation practices become less stringent. Clinicians should be aware of increases in some respiratory virus activity and remain vigilant for off-season increases. In addition to the use of everyday preventive actions, fall influenza vaccination campaigns are an important component of prevention as COVID-19 mitigation measures are relaxed and schools and workplaces resume in-person activities.

CDC analyzed virologic data* from U.S. laboratories available through the U.S. World Health Organization Collaborating Laboratories System^†^ (influenza only) and CDC’s National Respiratory and Enteric Virus Surveillance System^§^ (NREVSS) (multiple respiratory viruses). Reporting bias on the part of participating laboratories was minimized by requiring the following pathogen-specific inclusion criteria for noninfluenza viruses: 1) an average of ≥10 tests and ≥36 of 52 weeks of tests for RSV, RAdV, and HMPV or 2) ≥1 detection for each of the virus types for PIV (types 1–4) and HCoV (OC43, NL63, 229E, and HKU1). Hospitalization rates for influenza and RSV were calculated with data from the Influenza Hospitalization Surveillance Network (FluSurv-NET) and RSV Hospitalization Surveillance Network (RSV-NET).^¶^ Antigenic analyses for influenza viruses were conducted using hemagglutination inhibition or neutralization–based assays; viruses were tested for resistance to antiviral medications.** Influenza activity during October 3, 2020–May 22, 2021, and activity of other viruses during January 4, 2020–May 22, 2021 were described; data from 4 previous years were used for comparisons. Each date is the Saturday marking the week’s end.^††^ This activity was reviewed by CDC and was conducted consistent with applicable federal law and CDC policy.^§§^

During October 3, 2020–May 22, 2021, influenza activity was lower than during any previous influenza season since at least 1997, the first season for which data are publicly available (Figure 1) (Figure 2). Among 1,095,080 clinical specimens tested, 1,921 (0.2%) specimens were positive for an influenza virus: 721 (37.5%) for influenza A and 1,200 (62.5%) for influenza B. During this period, public health laboratories tested 502,782 specimens; 255 (0.05%) were positive for influenza, 153 (60.0%) were positive for influenza A, and 102 (40.0%) were positive for influenza B virus. Among 39 (25.5%) seasonal influenza A viruses subtyped, 18 (46.2%) were A(H1N1)pdm09 and 21 (53.8%) were A(H3N2). Of the 25 (24.5%) influenza B viruses with B lineage results, 17 (68.0%) were B/Victoria and eight (32.0%) were B/Yamagata. The cumulative incidence of laboratory-confirmed influenza-associated hospitalizations during this period was 0.8 per 100,000 (range = 62.0–102.9 during the previous four seasons). Five human infections with variant influenza A(H1N1)v, (H1N2)v, or (H3N2)v viruses^¶¶^ were reported from four U.S. states during the 2020–21 season. In each case, the patient reported direct contact with swine or living or working on a farm where swine were present before illness onset.

**FIGURE 1:**
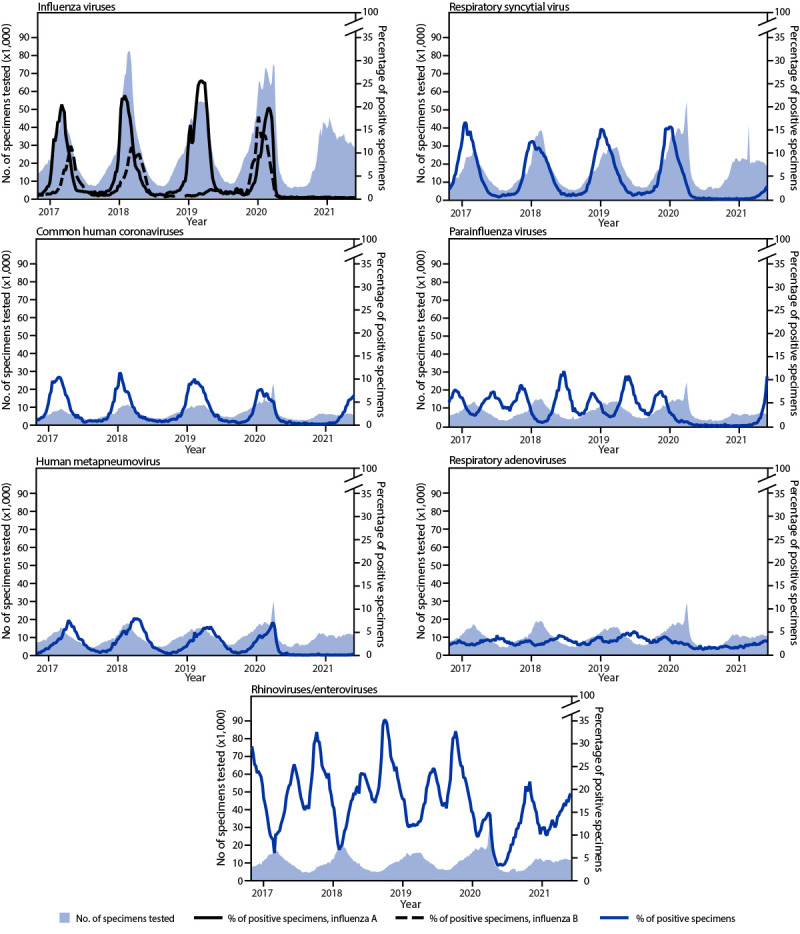
Number of specimens tested and the percentage of positive tests for influenza viruses, respiratory syncytial virus, common human coronaviruses, parainfluenza viruses, human metapneumovirus, respiratory adenoviruses, and rhinoviruses/enteroviruses, by year — United States, 2016–2021

**FIGURE 2:**
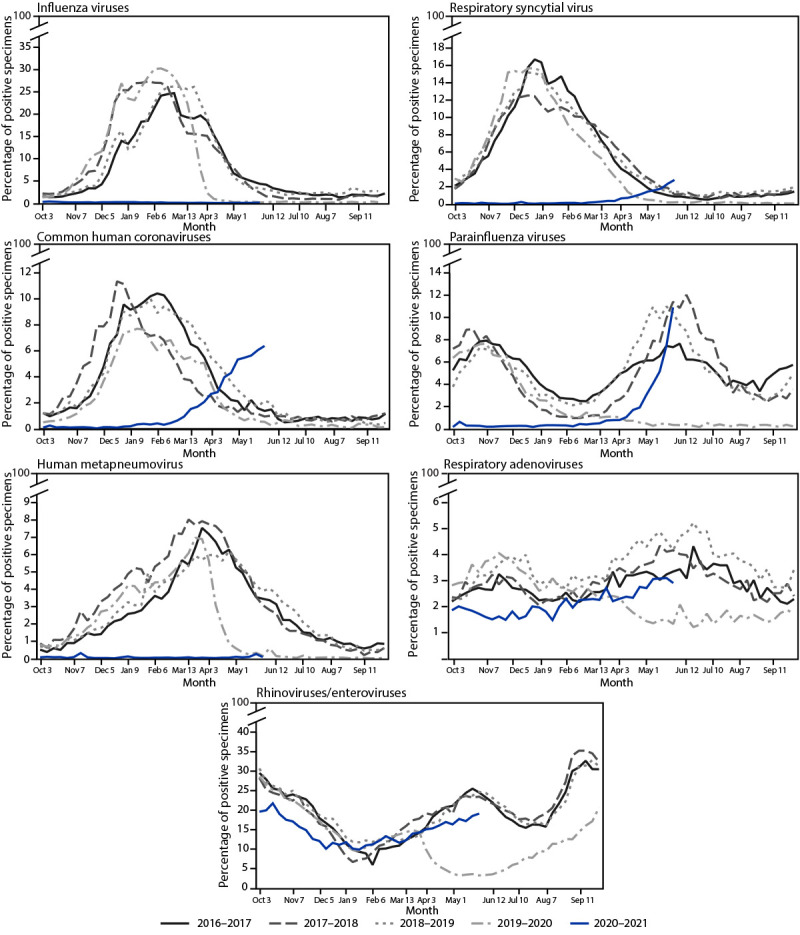
Percentage of specimens testing positive for influenza viruses, respiratory syncytial virus, common human coronaviruses, parainfluenza viruses, human metapneumovirus, respiratory adenoviruses, and rhinoviruses/enteroviruses, by month — United States, 2016–2017 through 2020–2021

Sixteen influenza viruses were genetically characterized. Phylogenetic analysis of influenza hemagglutinin (HA) genes indicated that of three influenza A(H1N1)pdm09 viruses, all HA genes belonged to the 6B.1A clade (two in 5A1 and one in 5B subclades); all five A(H3N2) viruses belonged to the 3C.2a1b2a subclade and all eight B/Victoria viruses belonged to the V1A.3 clade. Fifteen viruses were antigenically characterized by hemagglutination inhibition or virus neutralization-based methods. The three A(H1N1)pdm09 viruses were similar to the cell-based A(H1) component of the 2020–21 Northern Hemisphere influenza vaccines and two of these were also similar to the egg-based A(H1) component***; all eight B/Victoria lineage viruses were antigenically similar to the egg- and cell-based B/Victoria components of the vaccine. One of the four A(H3N2) viruses was similar to the cell-based A(H3) component of the vaccine (i.e., reacted within fourfold of homologous titer); none of the viruses were as antigenically similar to the egg-based component. All 10 viruses tested for susceptibility to therapeutics were susceptible to neuraminidase (NA) inhibitors and Baloxavir.

During January 4–April 4, 2020, the weekly percentage of positive RSV results decreased from 15.3% to 1.4%, then remained at historically low levels (<1.0% per week) for the next year (Figure 1) (Figure 2). During the previous 4 years, the weekly percentage of positive RSV results exceeded 3.0% beginning in October with peaks ranging from 12.5% to 16.7% in late December. During April 17–May 22, 2021, the weekly percentage of positive results increased from 1.1% to 2.8% (increases occurred predominantly within the southeastern United States in U.S. Department of Health and Human Services [HHS] regions 4 and 6^†††^). The cumulative incidence of RSV-associated hospitalization was 0.3 per 100,000 persons during October 2020–April 2021 (compared with 27.1 and 33.4, respectively during the previous two seasons); 173 (76.5%) of 226 RSV-associated hospitalizations reported during October 1, 2020–May 22, 2021 occurred in April and May 2021.

From January 2020 to January 2021, HCoVs and PIVs circulated at lower levels than during the preceding 4 years (Figure 1). From January 4, 2020 to April 18, 2020, the weekly percentage of HCoV-positive results declined from 7.5% to 1.3%, remained <1.0% until February 27, 2021, and increased to 6.6% by May 22, 2021 (led by types OC43 and NL63). During the previous 4 years, HCoV circulation peaks occurred during December–January and ranged from 7.7% to 11.4%. From January 4, 2020 to March 28, 2020, the weekly percentage of positive PIV test results decreased from 2.6% to 1.0%, then remained <1.0% until April 3, 2021, followed by an increase to 10.9% by May 22, 2021 (led by type PIV3). During the previous 4 years, PIV circulation peaked during the fall (October–November) and spring (May–June). The current increase could represent a return to prepandemic seasonality. From January 4, 2020 to March 14, 2020, the weekly percentage of HMPV positive results rose from 4.2% to 7.0%, dropped to 1.9% during the week of April 11, 2020, and remained <1.0% through May 22, 2021 (Figure 2). During the previous 4 years, HMPV circulation peaked between 6.2% and 7.7% in March and April.

From January 2020 to April 2021, the weekly percentage of RAdV positive results decreased to lower ranges (1.2%–2.6%) than those observed historically. The weekly percentage of positive results increased steadily to 3% by May 22, 2021, a level observed during previous surveillance years. The weekly percentage of positive RV/EV results declined from late March (14.9%) through early May 2020 (3.2%), levels lower than those typically observed during spring peaks (Figure 2). Weekly percentage of positive results then increased steadily until October 17, 2020, peaking at a lower level (21.7%) compared with fall peaks in previous years (median = 32.8%). In 2021, weekly percentage of RV/EV-positive results declined to 9.9% by January 16, 2021, before increasing to 19.1% on May 22, 2021; this could reflect the usual spring peak that has occurred in previous years (Figure 2).

## Discussion

In the United States, the circulation of respiratory viruses was disrupted during the COVID-19 pandemic, but the magnitude, timing, and duration of this effect varied among viruses. During 2020, influenza viruses and RSV circulated at historically low levels. In 2021, influenza continues to circulate at low levels whereas RSV activity has been increasing since April 2021, indicating an unusually timed increase in some regions of the country.^§§§^ HCoV and PIV activity is rising to prepandemic levels after notably low circulation, but this HCoV activity is inconsistent with the timing for a typical season. HPMV activity has remained low since March 2020. Although RAdV and RV/EV activity decreased in spring 2020, circulation has reverted to the week-to-week fluctuations at levels similar to those observed before the pandemic. Among each group of viruses, changes in the circulation of specific species and types warrant further assessment.

The duration of the effect of the COVID-19 pandemic and associated mitigation measures on respiratory virus circulation is unknown. Circulation of other respiratory viruses might continue to change as pandemic mitigation measures are adjusted and as prevalence of and immunity to both SARS-CoV-2, the virus that causes COVID-19, and immunity to these other viruses waxes and wanes. In 2020, influenza continued to circulate in the tropics; therefore, resumption of circulation in the United States is possible as global travel resumes (*3*). Every year, it is difficult to predict which influenza viruses might circulate during the next season (*4*). In the United States, influenza A (H3N2) viruses continue to be identified, but the diversity of the subclades co-circulating was reduced relative to recent seasons, and globally, few detections of influenza B viruses of the Yamagata lineage were detected during the pandemic. Reduced circulation of influenza viruses during the past year might affect the severity of the upcoming influenza season given the prolonged absence of ongoing natural exposure to influenza viruses. Lower levels of population immunity, especially among younger children, could portend more widespread disease and a potentially more severe epidemic when influenza virus circulation resumes. As the fall season approaches with schools and workplaces reopening, in addition to the use of recommended everyday preventive actions, clinicians should encourage influenza vaccination for all persons aged ≥6 months (*5*).

RAdV and RV/EV activity continued during 2020 and might be returning to prepandemic circulation patterns (*6*,*7*). Factors contributing to this distinct circulation are unclear but might include the relative importance of different transmission mechanisms, such as aerosol, droplet, or contact, the role of asymptomatic transmission, and prolonged survival of these nonenveloped viruses on surfaces, all of which might make these viruses less susceptible to nonpharmaceutical interventions, such as mask-wearing and surface cleaning (*8*,*9*). The delay in circulation of PIVs and HCoVs, which circulate at high levels among children, could be related to some schools suspending in-person classes until late winter. However, the relative absence of HMPV, which affects a similar age group as RSV (i.e., children aged <2 years) is unexplained. The unusual timing of rising RSV detections was also observed in Western Australia (*10*).

The findings in this report are subject to at least three limitations. First, changes in health-seeking behaviors during the pandemic (e.g., designated testing sites for COVID-19) might have contributed to a decrease in reported respiratory virus activity if routine health care visits were not made to health care providers who participate in surveillance. Testing for respiratory viruses was somewhat reduced during 2020–2021 but was higher than typically seen during periods of low virus activity. In addition, the detection of sporadic novel influenza viruses and increases in levels of circulation of other respiratory viruses attest to systems’ effectiveness. Second, each test result was independently reported, therefore the role of virus-virus interactions on activity could not be examined. Finally, some viral groupings (e.g., RV/EV) are large and might obscure type-specific patterns.

The different epidemiologic patterns of respiratory viruses observed during the COVID-19 pandemic in this U.S. surveillance summary raise questions about transmission and prevention, such as the contribution of birth cohort effects, natural immunity, and interventions. Clinicians should be aware that respiratory viruses might not exhibit typical seasonal circulation patterns and that a resumption of circulation of certain respiratory viruses is occurring, therefore an increased index of suspicion and testing for multiple respiratory pathogens remain important. Improved understanding of the role that nonpharmaceutical interventions play on the transmission dynamics of respiratory viruses can guide future prevention recommendations.

**Summary**

**What is already known about this topic?**

Nonpharmaceutical interventions introduced to mitigate the impact of COVID-19 reduced transmission of common respiratory viruses in the United States.

**What is added by this report?**

Influenza viruses and human metapneumovirus circulated at historic lows through May 2021. In April 2021, respiratory syncytial virus activity increased. Common human coronaviruses, parainfluenza viruses, and respiratory adenoviruses have been increasing since January or February 2021. Rhinoviruses and enteroviruses began to increase in June 2020.

**What are the implications for public health practice?**

Clinicians should be aware of increased circulation, sometimes off season, of some respiratory viruses and consider multipathogen testing. In addition to recommended preventive actions, fall influenza vaccination campaigns are important as schools and workplaces resume in-person activities with relaxed COVID-19 mitigation practices.

## Footnotes

* Influenza data as of July 7, 2021.

†
https://www.cdc.gov/flu/weekly/overview.htm

§ Some influenza clinical laboratory data and all other respiratory virus data are aggregate, weekly numbers of nucleic acid amplification tests and detections reported to NREVSS, a passive, voluntary surveillance network of clinical, commercial, and public health laboratories. NREVSS aggregate, weekly tests are reported specifically for each pathogen. NREVSS participating laboratories’ testing capabilities vary annually, and testing intentions vary for each pathogen. A range of 50–178 laboratories met the pathogen-specific criteria for inclusion criteria during a given surveillance year. https://www.cdc.gov/surveillance/nrevss/index.html

¶ FluSurv-NET and RSV-NET use similar methods; unadjusted cumulative incidence rates are calculated using CDC’s National Center for Health Statistics bridged-race postcensal population estimates for the counties included in the surveillance catchment area. Laboratory confirmation is dependent on clinician-ordered testing and cases identified through surveillance are likely an underestimation of the actual number of persons hospitalized with both pathogens. https://www.cdc.gov/flu/weekly/influenza-hospitalization-surveillance.htm; https://www.cdc.gov/ncezid/dpei/eip/eip-network-activities.html

** Genetic characterization was carried out using next–generation sequencing, and the genomic data were analyzed and submitted to public databases (GenBank or EpiFlu). Antigenic characterizations were carried out by hemagglutination inhibition assays or virus neutralization–based focus reduction assays to evaluate whether genetic changes in circulating viruses affected antigenicity; substantial differences could affect vaccine effectiveness. Testing of seasonal influenza viruses for resistance to the neuraminidase (NA) and polymerase inhibitors was performed at CDC using next-generation sequencing analysis, a functional assay, or both. NA sequences of viruses are examined for the presence of amino acid substitutions previously associated with reduced or highly reduced inhibition by any of the three NA inhibitors. https://www.who.int/influenza/gisrs_laboratory/antiviral_susceptibility/NAI_Reduced_Susceptibility_Marker_Table_WHO.pdf?ua

†† *MMWR* week numbers were used corresponding to week 40 in 2020 through week 39 in 2021. https://ndc.services.cdc.gov/wp-content/uploads/MMWR_Week_overview.pdf

§§ 45 C.F.R. part 46, 21 C.F.R. part 56; 42 U.S.C. Sect. 241(d); 5 U.S.C. Sect. 552a; 44 U.S.C. Sect. 3501 et seq.

¶¶ Phylogenetic lineage classification of variant swine viruses indicated that one A(H1N1)v influenza virus was reported from North Carolina and one A(H1N2)v was reported from Wisconsin. Each virus had an hemagglutinin (HA) gene closely related to the 1A.3.3.3 lineage of swine influenza virus. Another (H1N1)v influenza virus was reported from Iowa that had an HA gene derived from a seasonal A(H1N1)pdm09 virus that was likely introduced into swine by reverse zoonosis. In addition, an influenza A(H1N1)v virus was reported from a patient in Ohio. However, only partial HA and NA gene sequences could be obtained from the sample, thus no detailed lineage classification or antigenic characterization was possible. An A(H3N2)v influenza virus was reported from Wisconsin that had an HA gene closely related to H3N2 viruses currently circulating in the swine population, which was likely introduced into swine from humans in 2010.

***
https://www.who.int/influenza/vaccines/virus/recommendations/202002_recommendation.pdf

††† HHS *Region 4*: Alabama, Florida, Georgia, Kentucky, Mississippi, North Carolina, South Carolina, and Tennessee. *Region 6*: Arkansas, Louisiana, New Mexico, Oklahoma, and Texas.

§§§
https://emergency.cdc.gov/han/2021/han00443.asp
